# Risk Assessment of Histamine in Chilled, Frozen, Canned and Semi-Preserved Fish in Morocco; Implementation of Risk Ranger and Recommendations to Risk Managers

**DOI:** 10.3390/foods7100157

**Published:** 2018-09-25

**Authors:** Oleya El Hariri, Nourredine Bouchriti, Rachid Bengueddour

**Affiliations:** 1 Laboratory of Biochemistry, Biotechnology, Health and Environment, Department of Biology, Faculty of Science, University Ibn Tofail, Kenitra 14999, Morocco; rachidbengueddour@yahoo.fr; 2 Department of Pathology and Public Health Veterinary Unit HIDAOA, IAV Hassan II, Rabat 10112, Morocco; bouchriti@gmail.com

**Keywords:** histamine, risk assessment, fish, sardine, frozen, semi-preserved, canned, risk ranger, risk mangers, Morocco

## Abstract

A risk assessment of histamine was conducted for different categories of fishery products in the market. Risk estimates were assessed using the Risk Ranger tool. The estimated risks associated with the consumption of canned, semi-preserved and frozen fish are lower than those associated with fresh fish. According to the hypotheses of application or not of the histamine control measures, two risk levels were calculated for each product. The highest risk is associated to sardine with a score of 35 (equivalent to 39 patients per year). With the application of control measures, the score decreases to 20 (equivalent to one patient per year) with a reduction of 38 patients estimated per year. The risk ranking for frozen fish varies between zero (1 patient for 1000 years) and 11 (three patients for 1000 years). For semi-preserved fish, it ranges from zero to 21. For canned fish, the risk ranking varies between 12 (five patients for 1000 years) and 21 (15 patients for 100 years). As a result, most Moroccan seafood products are classified as “low risk”. However, it is recommended that risk managers maintain the adopted measures, strengthen interventions upstream in the food chain and that professionals maintain the HACCP (Hazard Analysis Critical Control Point) system effectively.

## 1. Introduction

Histamine is a biogenic amine that naturally occurs in the human body and has an important physiological role as neurotransmitter messenger [1]. There are two origins of histamine: the first is endogenous and the second exogenous by ingestion of certain foods such as fish and fermented products [2]. In healthy people, amine oxidase rapidly detoxify histamine, but consumers can develop severe symptoms of histamine intoxication if high amounts are ingested [2].

The incubation time for histamine intoxication is short, often ranges from 2 min to 2 h, and people usually develop symptoms while they are still eating. The main symptoms of histamine intoxication are cutaneous (rash, urticaria, oedema, and localized inflammation), gastrointestinal (nausea, vomiting and diarrhoea), haemodynamic (hypotension) and neurological (headache, tingling, oral burning sensation, flushing and itching) [3]. Symptoms are often mild, rarely severe [4] and frequently disappear after 24 h [5].

Histamine is formed in fish and fishery products due to certain bacteria capable of producing an enzyme, histidine decarboxylase. This enzyme will convert free histidine to histamine [4].

Temperature abuse during post-harvest chilling, storage and/or processing is one of the main factors influencing the production of histamine [2].

The main bacteria producing histamine are members of the family Enterobacteriaceae. However, Histamine is also produced, but to a lesser extent, by bacteria that can grow at refrigeration temperatures [3]. The implementation of hygienic measures and maintaining a cold chain at each step of the production and distribution are essential to avoid or limit the production of histamine.

European Regulation (EC) No. 2073/2005 of 15 November 2005 specifies fish families associated with high levels of histidine. These are Scombridae, Clupeidae, Engraulidae, Coryphaenidae, Pomatomidae and Scombrocidae [6]. A Joint Food and Agriculture Organization (FAO)/World Health Organization (WHO) Expert Meeting on the Public Health Risks of Histamine and other Biogenic Amines from Fish and Fishery Products presented a global list of fish species associated with scombroid poisoning [7].

Little scientific data concerning histamine intoxications are available in Morocco. During 2016, the official authority in Morocco recorded 2723 cases of food poisoning, of which 53.5% are collective cases. These intoxications represents 17.2% of all intoxication in Morocco, occupying third position.

Following the consumption of fish, five histamine-poisoning outbreaks were recorded in 2016 and 2 in 2017 [8].

A survey conducted in Morocco on fish consumption and frequency of fish poisoning showed that 68.5% of respondents had never encountered a poisoning problem and 28.2% had rarely [9].

Risk analysis is a process that has three components: risk assessment, risk management, and risk communication. Risk assessment is the scientific evaluation of known or potential adverse health effects resulting from human exposure to foodborne hazards [10]. The Codex Alimentarius risk assessment incorporates four steps which are hazard identification, hazard characterization, exposure assessment and risk characterization. The Codex states that risk assessment should be based on the most relevant national scientific data and should use the available quantitative and qualitative data [11].

Several tools and methods can be used to perform a risk assessment. The Risk Ranger is a risk calculation tool developed by the Food Safety Authority of Australia. It helps to determine the relative risks of various products/contaminants/treatments. It takes the form of a Microsoft Excel spreadsheet and incorporates principles of health risk assessment. It combines exposure probability to a food-borne hazard, magnitude of the hazard in a food if present, and severity consequences that may result from the exposure level and frequency [12].

Disease severity is affected by the intrinsic characteristics of the pathogen/toxin and a consumer’s susceptibility. Risk Ranger incorporates all factors that affect risk from a hazard in a particular product including:

- Severity of the hazard and susceptibility of the population of interest;
- The probability of a disease-causing dose of the hazard being present in a meal;
- The number of meals consumed by a population of interest in a given period [13].

The user is required to answer 11 questions related to hazard severity, the probability that a pathogenic dose of the hazard is present in a meal, and the probability of exposure to the hazard.

Risk Ranger uses risk assessment principles, as it incorporates the probability of exposure to a food risk, the prevalence of hazards in a food product where they exist, and the probability and the severity of the consequences of a level of contamination exposure.

The objective of this study is to assess the risk of histamine in the various categories of fresh or processed fishery products (canned fish, semi-preserved fish, and frozen fish) and to evaluate this risk following the consumption of various fish species known to contain high levels of histidine such as sardine, anchovy, mackerel and horse mackerel.

## 2. Materials and Methods

Risk Ranger is a spreadsheet that converts qualitative descriptions into numerical values and combines them with quantitative data in a series of mathematical functions [14]. This tool has been used for the various categories of produced and marketed fishery products that are associated with high levels of histamine production.

The worksheet converts qualitative inputs to numerical values and combines them with quantitative inputs through a series of mathematical and logical steps. These calculations are used to generate a risk ranking for public health [15]. Risk assessments for product-hazard combinations use a scale of 0 to 100. An increase of six in risk ranking is approximately a 10-fold risk increase [14].

To list the fish families associated with high levels of histidine, the [7] expert report was used and was compared with the list of the main species landed in various ports of Morocco. Sardines, anchovies, mackerel and horse mackerel are the most consumed fresh species by Moroccans and are the four species retained for the purposes of this study [9]. Seven major categories of fishery products were studied: refrigerated pelagic fish (sardine, anchovy, horse mackerel and mackerel), canned, semi-preserved and frozen pelagic fish.

## 3. Results and Discussion

The risk assessment of histamine under the Moroccan conditions by the Risk Ranger tool is based on the answers to each of the 11 questions. Risk Ranger is structured around three main chapters; each chapter contains a number of questions. The first two questions will evaluate the susceptibility and severity of the hazard; the third, fourth and fifth questions will judge the probability of exposure; and the rest of the questions will estimate the probability that food contains infectious or toxic dose.

### 3.1. Risk Ranger Questions

#### 3.1.1. Susceptibility and Severity

1. Question 1: Severity of the hazards

There are four levels varying according to the severity of the symptoms caused by this hazard. A difference in severity of 10 times exists between each hazard category.

- Hazard category “Severe” causes death or chronic sequelae in most cases, such as Tetrodotoxins and Botulinum toxin.
- Hazard category “Moderate” in most cases medical treatment is required such as *Listeria monocytogenes*, *Vibrio vulnificus* and *Vibrio cholerae*.
- Hazard category “Mild” where medical treatment is sometimes necessary like *Vibrio parahaemolyticus*, viral hepatitis, Norwalk virus, Ciguatera, *Salmonella* and histamine.
- Hazard category “Minor” where medical treatment is rarely required, such as *Staphylococcus aureus* and *Clostridium perfringens*.

The hazard of histamine is classified as a mild hazard by FAO [13] and by several scientific publications such as Lehane et al. [16]. In case of histamine intoxication, medical treatment is sometimes necessary. Taylor et al. [17] indicated that pharmacological intervention is not always necessary following histamine intoxication, as the disease is self-limiting and the symptoms last for a short time. Moreover, the competent authority of New Zealand has classified the risk of histamine poisoning as a low risk (Mild), and specifies that histamine occurs as a result of temperature abuse during the catch, transportation, storage and processing of fish species rich in free histidine [18]. Symptoms of histamine intoxication disappear rapidly after antihistaminic treatment [19].

Histamine is the toxin involved in histamine poisoning for several reasons:

- The symptoms of allergic reactions are typical to those caused by histamine and often appear within minutes of consuming the food concerned;
- Antihistamine therapy is usually effective and fast in less than eight hours;
- High levels of histamine are often found in fishery products that caused this reaction [20].

Histamine is formed following an enzymatic reaction of bacterial decarboxylation, which transforms histidine into histamine. The bacteria producing histamine are naturally present in the gills and intestine of fish and can spread to other tissues during handling [19].

The answer to this question is Mild.

2. Question 2: Susceptibility of the population of interest

There are four groups of populations that vary according to their levels of susceptibility to the various symptoms caused by this hazard and which are:

- Population category ”General population“ includes all members of the population;
- Population category ”Slightly susceptible” concerns young children (1 to 5 years old) and people over 65 years old. They are ranked 5 times more sensitive than the general population.
- Population category “Very susceptible”: this category includes new-borns, children under one year old and people with diseases such as diabetes, cancer and liver damage, which make them vulnerable to infectious diseases. They are classified 30 times more sensitive than the general population.
- Population category “Extremely susceptible” are people with acquired immunodeficiency (AIDS) or who are recovering from a transplant and have a very weakened immune system. They are 200 times more likely to succumb to risks than the general population.

Histamine poisoning can affect the general population of Morocco. Poisoning can occur in an adult as in a child or immunosuppressive [16]. The answer to this question is General.

#### 3.1.2. Probability of Exposure to Hazard in the Food Concerned

3. Question 3: Frequency of consumption

The higher the level of consumption, the greater the susceptibility to being affected by histamine gets. Various consumption frequencies are defined:

- Daily frequency;
- Weekly frequency;
- Monthly frequency;
- A frequency of a few times a year.

One study has shown that the frequency of fish consumption varies according to the various categories of fishery products and fish species [9]. The average frequency of consumption of fresh sardine is 1.74 per week, and 0.8 per week for canned fish. The frequency of consumption of semi-preserved fish is a few times a year (the average amount of consumption is 0.18 kg per year per person). The input data for this question took into consideration the results of this study, which reports consumption frequencies for the different categories of fishery products by the Moroccan consumer.

4. Question 4: Proportion of the population consuming the product

The proportion of the population that consumes the product is also a criterion to be considered. Four categories are set for the proportion consuming the product:

- All: if the entire population (100%) consumes this product;
- Most: if 75% of the population consumes this product;
- Some: if 25% of the population consumes this product;
- Very little: if 5% of the population consumes this product.

The results of the consumer survey conducted by [9] give us an idea about the pattern of fish consumption by Moroccans. The refrigerated sardine is consumed by 68.08% of Moroccans, fresh horse mackerel by 3.87%, fresh anchovy by 3.37% and mackerel by 1.25%. The proportion of Moroccans who consume canned sardines is 50.8%, while the percentages of Moroccans consuming semi-preserved and frozen fish are very low; they are 4% and 1.6% respectively.

5. Question 5: Size of the consuming population

The size of the consuming population is also a criterion to consider. According to the last census in Morocco carried out in 2014, the total number of the Moroccan population residing in Morocco is 33,762,036 inhabitants [21]

#### 3.1.3. Probability of Food Containing an Infectious Dose

6. Question 6: Probability of contamination of raw product per serving

This is the proportion of contamination of the given product by the considered hazard.

- Rare: a probability of contamination is classified as rare when the level of contamination is one per thousand;
- Uncommon: when the level of contamination is 1%;
- Sometimes: when the level of contamination is 10%;
- Common: when the level of contamination is 50%;
- All: when the level of contamination is 100%.

A study by [22] determined histamine levels in canned and semi-preserved fish marketed in Morocco since 2013–2015. The results showed that the occurrence of histamine is low. Of the 446 samples analysed, only 14 samples were non-compliant. The histamine average level of all the analysed batches was 5.63 mg/100 g, 5.14 mg/100 g for canned fish and 7.76 mg/100 g for semi-preserved fish. Moroccan legislation stipulates that for one lot to be acceptable, out of the nine samples taken independently of each lot, it is necessary that:

- The average histamine concentration is less than 100 mg/kg;
- A maximum of two samples based on the 9 may have histamine contents between 100 and 200 mg/kg;
- No sample should have a histamine content greater than 200 mg/kg.

Hence, these levels are well below the regulatory safety limit.

A summary of the official results of histamine in fresh pelagic fish marketed in the first sale Moroccan sites in 2018, showed that a histamine average level of 11.4 ± 4 ppm while no non-compliance was reported. These samples are taken by the inspectors of the competent authority in Morocco and concern pelagic fish such as sardine, anchovy, mackerel, horse mackerel, etc. Each sample is composed of nine units that are sent thereafter to official laboratories for analysis. Moreover, the synthesis of histamine results in fishery products for the years 2013–2015, carried out as part of Moroccan Food Safety Office’s official control in two official laboratories in Tangier and Agadir, is presented in Table 1.

This bibliographic review shows that the levels of contamination of the various fishery products manufactured and marketed in Morocco are very low.

The corresponding values of the contamination level of each product category will be integrated as responses to question 6.

7. Question 7: Effect of treatment

To answer this question, knowledge of the process and its effect on hazard is necessary. The various types of processes depending on their effects on hazard are:

- The process reliably eliminates the hazard;
- The process generally eliminates the hazard (99% of cases);
- The process slightly reduces (50% of cases) the hazard;
- The process has no effect on the hazard;
- The process increases (10×) the hazard;
- The process greatly increases (1000×) the hazard.

Enterobacteriaceae, e.g., *Morganella*, *Klebsiella* and *Hafnia* are the bacteria responsible for the production of histamine. Once histidine decarboxylase is produced, it can continue to produce histamine, even though bacterial growth has been prevented by cooling to 4 °C [16]. Ababouch et al. [24] showed that histamine production may increase even in ice storage. Furthermore, once a toxic level of histamine is reached, refrigeration has no effect on this level. According to [25,26], neither cooking, canning nor freezing reduces the toxic effect of histamine. When histamine is formed in a fish product, it will persist in the next steps.

Studies have shown that freezing fish can significantly reduce bacterial load and can limit the activity of decarboxylase enzymes produced prior to freezing [27]. Several authors have reported that when histamine is formed in fish, it is not inhibited by freezing or heating (in the case of normal cooking, hot smoking and canning) [26,28,29].

8. Question 8: Is there a recontamination potential after processing?

To answer this question, four categories for the possibility of recontamination after treatment exist

- NO;
- YES—minor (a frequency of 1%);
- YES—major (a frequency of 50%);
- OTHER.

The treatments for the products concerned by our study are sterilization for canned fish, salting for semi-preserved, freezing for frozen fish, and refrigeration for fresh fish.

1. **The canned fish:** sterilization is an effective process since it can destroy most of the bacterial flora. Therefore, no increase in histamine levels is observed then, there is no possibility of recontamination after sterilization. The answer is “No”. Canned fish can only be considered safe for recontamination if it is immediately consumed after opening. Many cases of scombroid poisoning have been reported in Italy after eating canned tuna stored open. A common practice of selling scombroid products (mackerel, tuna and anchovies) on the basis of large open cans, often for several days [30].
2. **Frozen fish:** the freezing treatment inhibits the growth of bacteria. In this case, there is no possibility of recontamination after freezing, thus no increase in histamine levels is detected and the answer will be “No”.
3. **Semi-preserved and fresh fish**: there is a possibility of recontamination if the cold chain breaks.

▪ ***For Semi-Preserved Fish***

Semi-preserved fish are products that have undergone a maturation treatment and has led to several physico-chemical and biochemical changes. Semi-preserved are products characterized by a pH of 5.3 to 5.7, a water activity (Aw) of less than 0.76, a moisture level of less than 50% and a chloride rate (NaCl) of more than 15%. These parameters associated with storage at a temperature of 15 °C create conditions that allow a better protection against possible bacterial growth [31]. Hence, if this treatment is well conducted, the production of high levels of histamine can be limited.

However, authors have reported that in the case of temperature abuse, high levels of histamine may be produced. The role of *Pediococcus halophilus* in the production of histamine has been highlighted by [32]. This bacterium is able to decarboxylate histidine even at a concentration of 20% salt in the anchovy muscle and it becomes predominant after the third month of maturation. This species is strictly mesophilic and capable of producing histamine during storage at room temperature (20 °C) (the optimum temperature is 30–35 °C), and is inhibited when temperature is less than 15 °C [33].

The evolution of biogenic amines in marketed semi-preserved anchovies was studied [34]. They reported that histamine production was observed after 6 months of storage at 18–22 °C and that no increase in histamine was observed during storage at refrigeration temperatures (4–6 °C).

A recent study on salted shad demonstrated that the properties of this product (a salt content of 10% and a water activity (aw) of less than 0.75) were not effective in inhibiting the production of histamine during storage at room temperature (18–22 °C). The bacteria responsible for the production of histamine are mainly halotolerant bacteria [35].

At this level, two assumptions will be submitted:

- If good hygiene practices and an adequate, the HACCP (Hazard Analysis Critical Control Point) system is applied, the level of cross-contamination is “No” (A frequency of 0%).
- If an establishment has not followed good practice: by referring to question six, the level of cross-contamination found is low and does not even reach 1%. In addition, most establishments in Morocco set up a HACCP system. Therefore, post-treatment contamination is controlled and a minor potential for recontamination is possible (with a frequency of 1%).

▪ ***For Fresh Fish***

Highly histamine-producing bacteria are mesophilic. Several authors have reported the significant inhibition of histamine formation in fish stored at low temperatures [17]. A study by [36] found histamine levels of 78.9 and 384.4 mg/100 g, respectively, after 12 and 24 h of storage at room temperature. In another test, the histamine content was 25.9 and 86.7 mg/100 g, respectively, after 16 and 32 h of storage at room temperature. It was found by [24] that levels of 235 mg/100 g of histamine are reached after 24 h of storage at room temperature. Marrakchi et al. [37] found that histamine levels were 30 mg/100 g in Moroccan sardines (*Sardina pilchardus*) stored at room temperature (25 °C) for 24 h. In another study, a histamine content of 84 mg/100 g after 12 h and 147.9 mg/100 g after 24 h of storage at room temperature was detected [38].

The kinetics of histamine formation during fish storage is characterized by two phases:

- A first long phase with little or no histamine production. This phase corresponds to the time required for the histamine-producing bacteria to multiply and reach high levels. The importance of this stage depends mainly on the initial bacterial concentration, their growth rate and temperature.
- A second phase where the level increase rapidly. The rate of histamine formation during the second phase corresponds to the activity of high concentrations of histamine-producing bacteria, and is influenced by the storage conditions and the characteristics of the product [39].

Some authors have reported that even when stored at low temperatures, histamine is always produced, but at generally lower levels than when stored at room temperature (25 °C) [40]. It was reported by [24] that changes in histamine levels are much slower for sardines stored in ice than at room temperature. Histamine reached toxic levels after 6 days at 8 °C [41,42].

It was shown by Fletcher et al. [43] that histamine-producing bacteria must generally reach a level greater than 10^7^/cm^2^ to produce histamine levels >5 mg/100 g. It was revealed by [36] that the initial load of mesophilic bacteria in sardine stored at room temperature is 3.4 × 10^3^–4 × 10^4^ CFU/g and became 3.8 × 10^6^–8 × 10^7^ CFU/g at the time of discharge. This charge is 1.2 × 10^3^–5 × 10^4^ CFU/g in ice storage and became 2 × 10^6^–7 × 10^7^ CFU/g at the point of rejection [36]. The bacterial load in sardine, responsible for the production of histamine, is low. Of the 568 bacterial isolates, only 55 are histamine producers. Microbiological tests identified 51 of 55 isolates such as Enterobacteriaceae, 35 of which were *Proteus* sp., seven isolates were *Morganella morganii*, two isolates were *Proteus vulgaris*, one isolate was *P. mirabilis*, 20 isolates unidentified *Proteus* species and four unidentified isolates [40]. It was found by [32] that the proportion of histamine-forming bacteria represents less than 1.37% of isolates of the total flora. The FAO has reported that a level of 10^8^/cm^2^ is the number of bacteria required for fish to contain toxic levels of histamine [13] and this is the rate adopted for our study.

Therefore, the initial bacterial load in histamine-producing bacteria is very low in sardine.

Two hypotheses are present at this level:

- In the case of compliance with good hygiene practices, the level of cross-contamination is No (A frequency of 0%).
- In the case of rupture in the cold chain and a lack of respect for good practices, a minor recontamination potential can occur (with a frequency of 1%).

9. Question 9: How effective is post-treatment control?

The answer to this question is an assessment of handling conditions during storage, distribution and retail. Risk Ranger provided four categories depending on the effectiveness of post-treatment control:

- Well controlled: when a reliable and effective system is set up, so no increase in pathogen or accumulation of toxin or microorganism occurs.
- Controlled: when a generally reliable and efficient system is put in place, so that there is little multiplication or accumulation (by 3).
- Not controlled: when there is no system (untrained staff) (increase of 10 times).
- Abusive gravity: a large increase of pathogens (1000 times).
- Non-pertinent: the level of hazard does not change.

In Morocco, the implementation of HACCP in fishery establishments is a regulatory obligation under law 28-07 on the food safety [23]. This system allows establishments to continuously implement preventive and corrective measures of all hazards (including histamine) that can compromise the quality and safety of products from raw material reception to final product expedition.

According to a study, between 2005 and 2015, 415 RASFF (Rapid Alert System for Food and Feednotifications for histamine in fishery products exported from various countries of the world to the European market were registered. The maximum number of notifications concerned fresh fish (*n* = 165), followed by canned fish (*n* = 123), frozen fish (*n* = 75), semi-salted fish (*n* = 14), fish sauce (*n* = 11) then dried fish (*n* = 10) [44]. The fish species with the highest level is tuna (1061.25 mg/kg).

For Morocco, during these 11 years, the average number of RASFF notifications for histamine was 5 per year. The highest number of notifications was recorded in 2011 and 2012 (*n* = 14). In this period, the Moroccan competent authority and professionals took drastic measures to regain histamine control. This control finds its justification in the decrease of the number of notifications the following years. The Moroccan products concerned by these notifications are canned, frozen and semi-preserved fish. This study also showed that most of the frozen fish notifications concerned products originating from southern Morocco (111.6 tons were discarded). The maximum quantity of canned originating from Safi (127.583 tons) followed by Agadir (121.42 tons), while the semi-preserved products were from Agadir.

It is recommended that the competent authority and professionals further strengthen the measures for histamine control in these areas. However, the study showed that the quantities rejected for histamine reasons represent 0.014% of the total quantity exported; hence, the study considered this rate to be low.

Another study by [22] has determined the levels of histamine in canned and semi-preserved fish in the Moroccan market from 2013 to 2015. This study presents the results of the histamine control plan of 446 samples of fish products, taken from various areas of Morocco, from markets, supermarkets, commercial and social catering. The synthesis of the data showed that the occurrence of histamine is low.

In Morocco, the transportation of perishable food products is regulated. The law requires sanitary approval. National Food Safety Office (ONSSA) inspectors issue this agreement after a regular check of sanitary, hygienic and technical conditions allowing the production and maintenance of the regulatory temperatures [23].

Regarding the upstream of the fish sector, Morocco has adopted several upgrading strategies, such as the implementation of specific good hygiene practices guides for fishing vessels and for the use of ice [45,46] Another strategy consists of the generalization of the use of standardized containers in all Moroccan ports designed to preserve the fish quality and to prevent cross-contamination. Morocco has implemented another program to upgrade coastal and artisanal fishing vessels. The “IBHAR” program provides financial support to ship-owners to replace old boats with new prototypes made of steel or other material rather than wood. The subsidized actions also concern the isolation and refrigeration of holds and the installation of ice machines [47]. These actions undertaken by the state have been of great importance to improving not only the commercial quality of fish but also to preserving their sanitary quality especially in terms of histamine. However, additional efforts must be made, especially by increasing the number of ice factories that can be subsidized by the state in order to facilitate the access to ice to all users, and therefore achieve a better control of histamine.

Catching has an effect on the production of histamine. If a pelagic fish is well iced on board after capture, no production of high levels of histamine will occur while in the case of none or insufficient refrigeration, histamine-producing bacteria will produce histamine depending on the abuse of temperature and time.

Two situations will occur:

▪ ***Good Refrigeration on Board***

FSSP software (Food Spoilage and Safety Predictor, Technical University of Denmark, Kongens Lyngby, Denmark) predicts the growth of microorganisms in food. It contains an application that predicts histamine levels and microbial load depending on temperature and storage time.

**Figure 1:**
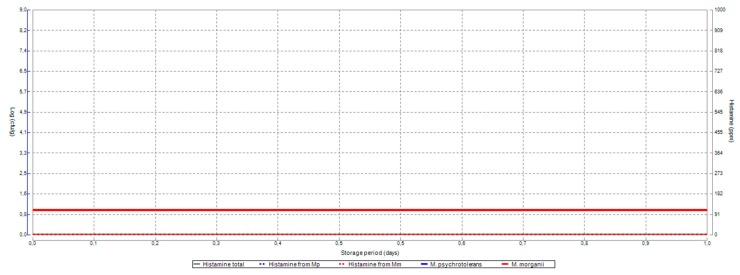
Application of Food Spoilage and Safety Predictor (FSSP) in the case of a good control of temperature.

In a case where refrigeration is sufficient and precocious, histamine contents and the microbial load remain low throughout the fishing period (Figure 1).

When fish is caught and stored in sufficient quantities of ice or any other refrigeration medium, the growth of mesophilic bacteria will be inhibited. Therefore, no production of histidine decarboxylase enzyme or large levels of histamine will be observed. In this case, the process is well controlled and then the answer for question 8 will be “well controlled”.

▪ ***Insufficient Refrigeration on Board***

**Figure 2:**
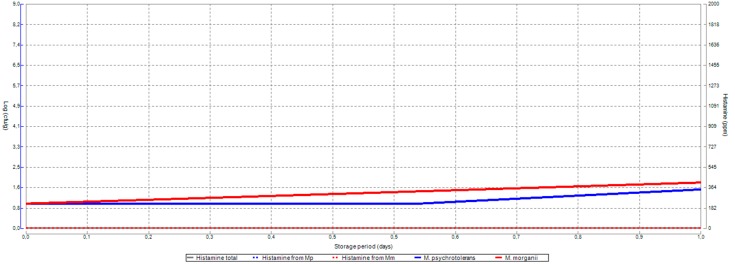
Application of FSSP in the case of insufficient icing.

If fish is not well refrigerated after catching and only insufficient quantities of ice are used, fish temperature will decrease at the beginning of refrigeration. However, as the fishing time increases, the temperature of the product will increase gradually following the slow ice melting. This early refrigeration will prevent the rapid growth of histamine-producing bacteria especially mesophilic ones. The result will be a low production of histidine decarboxylase enzymes and histamine (Figure 2).

The fishing time in Morocco for pelagic fish is generally short and fishing takes place during the night because of the nocturnal migrations of these pelagic fish towards the surface. Ambient temperatures at night are low compared to those recorded during the day. Besides, fish are usually cooled immediately after landing and the temperatures will be quickly reset to zero, thus the bacteria will be inhibited and the risk of producing high levels of histamine is prevented. A set of control measures to prevent the production of high levels of histamine must be reinforced by others.

The FDA has proposed secure fishing times depending on fish temperature. Indeed, the FDA recommends that fish be placed in ice or sea water chilled at 4.4 °C, at least within 12 h of fish death or at 10 °C within 9 h. These durations will be a good management measure in the absence or insufficiency of refrigeration [26].

Additional studies of the secure fishing time in the absence of refrigeration should be implemented following Moroccan climatic, geographical and seasonal conditions. These studies must establish the secure fishing time for all fish species that can have a high level of histidine.

A study showed that only some consumers (12.1%) use coolers, but 86.7% use plastic bags when buying fish. This is a risky practice, but fortunately, it is diminished by the fact that 85.2% of respondents buy their fish on the market near their homes. The main practice adopted by Moroccans after purchasing fish is to clean it immediately and consume it the same day (65%). Eight percent of respondents clean the fish and freeze it for later use and 7% adopt the same practice but without prior cleaning. This latter behavior increases the risk of histamine poisoning. In fact, during unwell thawing, the bacteria present in the viscera will migrate to the flesh of the fish, multiply and cause production of high levels of histamine; 81% and 70.3% of respondents are aware of the importance of the cold chain and the good hygiene practices respectively whilst 19% and 7% of them respectively were not aware [9].

Regarding the preference of respondents about culinary habits, the majority of Moroccans 49.5% prefer to prepare fish as *Chwaya* (grilled traditionally) which needs fresh fish caught on the same day, a culinary practice that prevents the risk of histamine [9].

Based on the above, two hypotheses will be assumed in this case; a post-treatment control level “well controlled”, and a second hypothesis “controlled”.

10. Question 10: What level of increase is needed to cause disease?

The level of bacterial growth associated with histamine levels harmful to human health is 10^8^ [13]. The initial number of histamine producing bacteria according to the answer to question 6 is low. Therefore, the growth required for bacteria to produce toxic histamine levels varies between 10^6^ and 10^7^ bacteria per g for all fish categories [13].

The Risk Ranger tool provided four choices, which are:

- None;
- Light (10 times increase);
- Moderate (increase of 100 times);
- Significant (10,000 increase).

There will be two assumptions:

- The case of canned, semi-preserved, frozen fish and fresh fish if the cold chain is respected: A significant increase of the germs is necessary to produce histamine intoxications (increase of 10^7^).
- Perishable products requiring cold control: if the cold chain is broken, a significant increase in germs is required to produce histamine intoxication (increase of 10^6^).

11. Question 11: Effect of meal preparation

This question deals with the effect of cooking meals on histamine. Cooking treatment does not destroy histamine formed in fish. Histamine is thermostable; then the answer selected is “No effect”.

**Figure 3:**
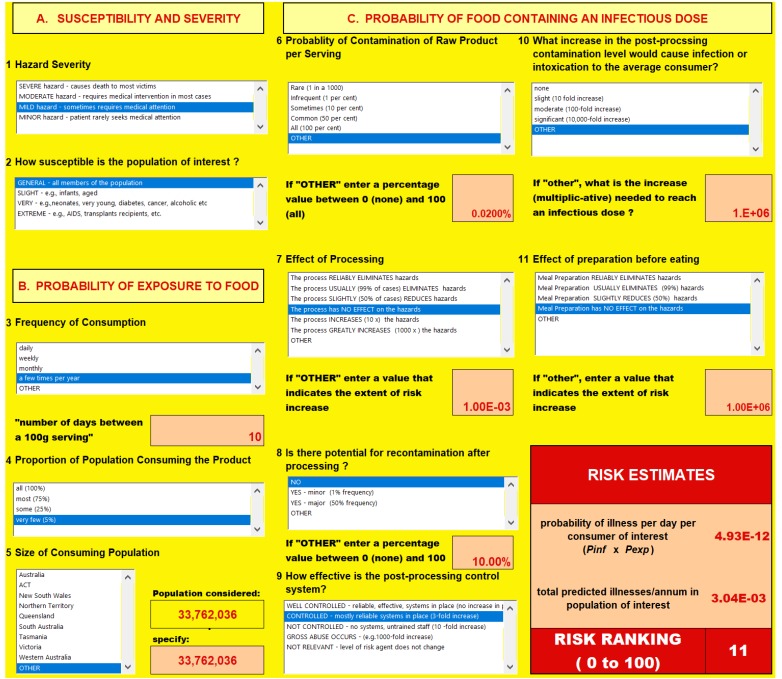
Screenshot for frozen fish.

Figure 3 shows a screenshot of the risk ranger interface and answers to the 11 questions for frozen fish.

### 3.2. Risk Estimates for Histamine

The Risk Ranger tool combines the factors of the answers to the 11 questions and generates three risk estimates:

- A risk ranking by a score between 0 and 100;
- The number of diseases expected annually in the selected population;
- The probability of illness per day in the target population.

**Figure 4:**
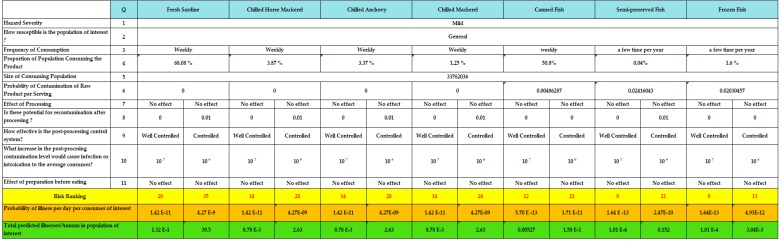
Summary of Risk Ranger applications for different categories of fishery products.

Figure 4 summarizes the applications of the Risk Ranger tool for all categories of fishery products and various fresh fish species with various inputs and outputs such as risk ranking, the projected number of patients per year and the probability of illness per day in the target population.

Two results corresponding to the various assumptions are proposed for each product group. A proposal corresponding to a low risk estimate (no recontamination, significant bacterial growth is required to reach the level associated with a histamine concentration), and a “higher” estimate (high prevalence of recontamination after treatment and low bacterial growth are necessary to reach the level associated with a high concentration of histamine).

**Figure 5:**
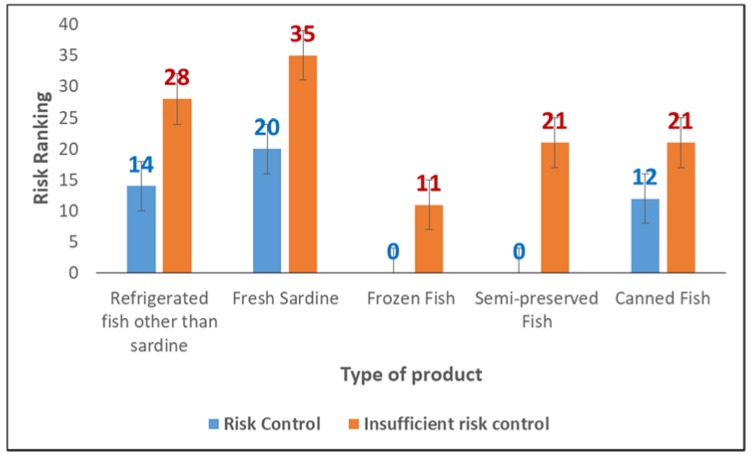
Risk ranking of various categories of fishery products (low- and high-risk ranking of histamine).

The estimated risks associated with the consumption of canned, frozen and semi-preserved fish are lower than the risk associated with the consumption of fresh fish (Figure 5).

The risk associated with canned fish varies between 12 and 21, risk associated with semi-preserved is from zero to 21, and the one associated with frozen fish is from zero to 11. Fresh fish varies from 20 to 35 and from 14 to 28 for sardine, and for other pelagic fish species, respectively.

The lowest risk score is zero and is associated with the consumption of semi-preserved and frozen fish in the case of histamine risk control, while the highest score of 35 is related to the consumption of fresh sardines in the absence of control measures.

When the risk of histamine is controlled, the risk score for sardine is 20, which corresponds to a single expected case of poisoning per year. Otherwise, the score is 35 corresponding to an estimated 39 cases of histamine intoxication expected in one year.

In the case of the implementation of control measures, following the consumption of canned food, the estimated number of patients per year is 5.27 × 10^−3^, whereas in the opposite case, the number is 1.58 patients per ten year.

The calculation of the risks shows that estimates of the number of patients per year can be reduced as it can be multiplied (Table 2). Risk management for canned and fresh fish has reduced the number of patients per 100 per year. The key to controlling histamine is the respect of the cold chain to inhibit histamine production, the respect of good hygienic practices to prevent cross-contamination and the rigorous application of HACCP system in processing establishments.

Risk benchmarks for Risk Ranger are:

- Low risk: risk score below 32;
- Moderate risk: risk score from 32 to 48;
- High risk: risk score higher than 48.

Referring to these scores, the various categories of products subject of our study are classified in the low risk score with the exception of fresh sardine, which is classified as moderate risk when histamine is not controlled.

Consequently, under current Moroccan conditions, the consumption of all fishery products (canned, frozen, semi-preserved and fresh fish) does not constitute a high risk of histamine for Moroccan consumers. Consumption of fresh sardines associated with a lack of control of histamine constitutes a moderate risk in Morocco. Risk managers need to focus more on the implementation of management and control measures for fresh sardines.

From 2010 to 2016, 364 cases of allergic diseases have been recorded in Morocco; 0.6% are due to fish consumption. The distribution of fish products responsible for poisoning is as follows: 2% are frozen fish, 2% canned fish, 68.75 fresh fish, 18% sardine and 4% other fish species such as sardine. This data is in harmony with the results of this study.

In addition, in 2016 and following fish consumption, five outbreaks of histamine intoxication were recorded and two in 2017. Rates that remain low and are consistent with the result of this risk assessment. The risk of histamine in Morocco is low. A study evaluating the risk of histamine, carried out in France using this Risk Ranger tool, found a score of 23 to 28 for canned, 25 to 37 for chilled fish, 22 to 33 for chilled processed products and 20 to 31 for fresh salmon [14]. In Australia, the risk assessment of histamine had 40 as a risk score for the general population [13].

Currently, the risk for the majority of fish products marketed in Morocco is moderate, but it is recommended that Moroccan risk managers rely on this risk assessment to maintain the currently adopted measures and to strengthen management measures by acting more effectively upstream of the food chain. Management measures are:

- Increasing capacity and installing new ice factories in all Moroccan ports;
- Subsidizing ice by the state, to make it financially accessible for all wholesalers and fishermen;
- Multiplying the training of fishermen on good hygiene practices and the respect of cold;
- Limiting fishing time to avoid producing high levels of histamine if ice is lacking;
- Strengthening control and inspection along the food chain, especially at the upstreaming of the chain;
- Strengthening the official control of the marketing of fresh fish, especially with respect to the cold chain;
- Increasing the number of consumer awareness campaigns;
- Requiring fish-processing establishments to draw up specifications with fishing vessels for good control of histamine;
- Designing small boats to carry larger amounts of ice (design the fisherman’s seat as hermetic containers);
- Sensitizing fish sellers to good hygienic practices;
- Planning studies that aim to find the secure time fishing depending on water and air temperatures, and on the size and fish species, in order to secure a good control of histamine;
- Raising awareness of boat owners of the importance of ice to preserve not only the marketability but also the safety of fish. The main messages that can be transmitted are:
  - Fish must be refrigerated immediately and early once caught;
  - Avoid mishandling, overloading and excessive piling of fish, as crushing fish accelerates the spread of histamine-producing bacteria from the intestines, gills and skin to fish muscles;
  - Use enough ice to completely wrap the fish to bring the internal temperature of the fish below 4 °C as soon as possible after capture to slow down bacterial growth and enzyme activity;
  - The catch volume must not exceed the cooling capacity to be achieved and must maintain the required temperatures.

Further research is needed to study the evolution of histamine-producing bacteria in sardine from capture to consumption in order to have an accurate record of the rate of increase required to reach the toxic histamine dose. Monitoring the temperature of the fish on the boat during a fishing trip is also expected.

## 4. Conclusions

The results of this study will be a basis for helping risk managers prioritise management actions and decide on which first interventions to make. The risk of histamine poisoning of Moroccan consumers following the consumption of canned, frozen and semi-preserved fish products is lower, compared to that which may occur following the consumption of fresh sardine or other fresh pelagic fish.

With respect to the cold chain, the application of good hygiene practices are the key to reducing the number of people intoxicated by histamine per year.

Recommendations have been sent to risk managers. A good control of histamine must take its starting point in the upstream of the food chain and exactly at the moment of catching. The best ways to prevent histamine formation is the rapid cooling of fish immediately after catching, and the application of good hygiene practices along the food chain from the boat to the consumer’s table.

## Figures and Tables

**Table 1:** Summary of histamine analyses in fishery products [23].

Nature of Products	Analyses Number	2013	2014	2015	Total	Rate of Non-Compliance (%)
Canned fish	Realized	21,459	15,203	4466	41,128	0.0049
	Non-Compliant	0	2	0	2	
Semi-preserved fish	Realized	14,985	15,057	7209	37,251	0.024
	Non-Compliant	5	2	2	9	
Fresh fish	Realized	18	2	54	74	0
	Non-Compliant	0	0	0	0	
Frozen fish	Realized	15,672	11,916	1962	29,550	0.020
	Non-Compliant	3	0	3	6	
Total	Realized	52,134	42,178	13,691	108,003	0.016
	Non-Compliant	8	4	5	17	

**Table 2:** Estimated risk reductions for each product category.

Product	Score Minimum	Score Maximum	Calculation of Rank Reduction	Risk Reduction Estimate (Number of Patient/Year)
Refrigerated fish other than sardine	14	28	14	100
Refrigerated sardine	20	35	15	100
Canned fish	12	21	9	100
Semi-preserved fish	0	21	21	1000
Frozen fish	0	11	11	10
